# Chromosome Organization in Early Meiotic Prophase

**DOI:** 10.3389/fcell.2021.688878

**Published:** 2021-06-03

**Authors:** Corinne Grey, Bernard de Massy

**Affiliations:** Institut de Génétique Humaine, Centre National de la Recherche Scientifique, Université de Montpellier, Montpellier, France

**Keywords:** meiosis, HiC, synapsis, loops, DNA double-strand breaks, recombination, prophase, cohesin

## Abstract

One of the most fascinating aspects of meiosis is the extensive reorganization of the genome at the prophase of the first meiotic division (prophase I). The first steps of this reorganization are observed with the establishment of an axis structure, that connects sister chromatids, from which emanate arrays of chromatin loops. This axis structure, called the axial element, consists of various proteins, such as cohesins, HORMA-domain proteins, and axial element proteins. In many organisms, axial elements are required to set the stage for efficient sister chromatid cohesion and meiotic recombination, necessary for the recognition of the homologous chromosomes. Here, we review the different actors involved in axial element formation in *Saccharomyces cerevisiae* and in mouse. We describe the current knowledge of their localization pattern during prophase I, their functional interdependence, their role in sister chromatid cohesion, loop axis formation, homolog pairing before meiotic recombination, and recombination. We also address further challenges that need to be resolved, to fully understand the interplay between the chromosome structure and the different molecular steps that take place in early prophase I, which lead to the successful outcome of meiosis I.

## Introduction

Meiosis is a specialized cell cycle in which diploid cells are converted into haploid cells.

During meiosis, diploid cells proceed through an S phase, also called premeiotic S phase, and then enter an extended prophase to reach the first division or meiosis I. The unique mode of chromosome segregation at meiosis I, called reductional segregation, requires the establishment of connections between homologous chromosomes (homologs) to allow their proper alignment and separation (Hunter, 2015). Multiple events occur during prophase I to allow the interaction between homologs and the formation of at least one crossing over (CO) per homolog pair, by homologous recombination. At the DNA level, exchanges are highly regulated in time, space, and choice of recombination partner. The homologous recombination pathway is initiated by the formation of DNA double-strand breaks (DSBs) at the onset of prophase I (i.e., leptotene) in most species, and their repair is completed at the end of pachytene. DSBs are not randomly distributed along the genome, and the choice of the sister chromatid or the homologous chromosome during their repair is regulated. At the chromosomal level, the pairing process allows each homolog to find and interact with its partner, and recombination (i.e., DSB formation and repair) stabilizes the interactions through non-reciprocal and reciprocal exchanges. This process is ensured in parallel for all chromosome pairs within the meiotic nucleus (Zickler and Kleckner, 2015). Cytology was crucial for identifying the connections between homologs that were named chiasma by Janssens in 1909 (Janssens et al., 2012). Since then, a large number of cytological studies have described and analyzed the chromosomal architecture and organization during meiotic prophase I, particularly the specific loop-axis organization of meiotic chromosomes that appears at prophase I onset, after the premeiotic S phase (Zickler and Kleckner, 1999), and the specific anchoring of telomeres to the nuclear envelope (Klutstein and Cooper, 2014). Both features are dynamic during prophase I, and play important roles in recombination and prophase progression, and thus in the proper execution of meiosis I.

**FIGURE 1:**
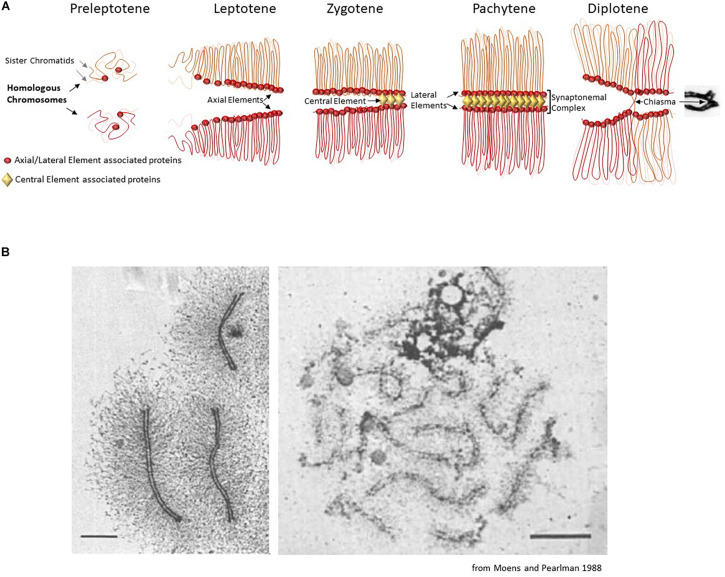
**(A)** Schematic description of the chromosomal organization during the first meiotic prophase. The first meiosis-specific proteins of the axial element are loaded on chromosomes during meiotic S-phase at the preleptotene stage. At leptotene stage, the elongating axial elements serve as scaffolds along which sister chromatids attach and begin to form an array of loops. At the zygotene stage, the proteins of the central element start to load and to initiate the formation of the synaptonemal complex that associates homologous chromosomes (homologous synapsis). In most organisms, homologous synapsis depends on meiotic DNA double-strand break formation and repair. During the pachytene stage, homologous chromosomes are fully synapsed and the chromatin is organized in a tight loop-axis array. Meiotic double-strand break repair is completed during this stage. The proteins of the central element unload and the synaptonemal complex dissolves during the diplotene stage, while homologous chromosomes remain connected only at centromeres and at chiasmata (the cytological manifestation of crossing-over sites). **(B)** Chromosome loops and axis visualized by electron microscopy. Left panel: chromosome spread of a pachytene nucleus in the moth *Hyalophora columbia*. The chromatin loops extend about 3 μm from the parallel-aligned chromosome cores, which together form the synaptonemal complex. Scale bar = 2 μm. Right panel: chromosome spread of a pachytene nucleus in *S. cerevisiae*, presenting a smaller loop size to axis ratio. Scale bar 2 μm.

The loop-axis organization can be observed in early prophase I, by electron microscopy and immunocytochemistry in many species, where two adjacent sister chromatids are organized as an array of loops anchored to a proteinaceous axis (Zickler and Kleckner, 1999; Figure 1). Several proteins are part of this axial structure: the cohesin complex(es) (Ishiguro, 2019), type II DNA topoisomerase (TopoII) (Moens and Earnshaw, 1989; Klein et al., 1992), condensins (Yu and Koshland, 2003; Mets and Meyer, 2009; Wood et al., 2010; Lee, 2013), and other proteins that are expressed specifically in meiotic cells (described below).

This review presents the current knowledge on the organization of meiotic chromosomes at the onset of meiotic prophase when the axial structure, also called the axial element, forms and before it engages into interaction with the homolog where additional structural components come into play for the formation of the synaptonemal complex (Figure 1). We present the associated proteins, how they contribute to this organization, and their roles in the execution of the meiotic recombination program during meiotic prophase.

The first part describes the knowledge gained in *Saccharomyces cerevisiae* where the identified proteins and functions provide a framework for understanding this organization. Then, it focuses on the main currently known players, the cohesin complex and the axis proteins Hop1 and Red1. This is followed by the second part that presents data obtained in mammals on the proteins that build and organize meiotic chromosomes with a detailed description of the best characterized components: the cohesin complexes and HORMAD1 and SYCP2 (orthologs of *S. cerevisiae* Hop1 and Red1, respectively). Then, the various identified or postulated functions of these proteins in the initiation of the meiotic recombination program are discussed. Insights gained from other species that provide complementary information from those obtained in yeast and mammals are included, to outline the evolutionary conservation of this functional organization among eukaryotes.

## Organization of the Chromosome Axial Element in *S. cerevisiae*

In *S. cerevisiae*, genetic and cytological studies allowed identifying the key components of meiotic chromosomes and their roles, among which the meiotic cohesin complex and the Hop1/Red1 axis- associated proteins are central players.

### The Meiotic Cohesin Complex, With the Specific α-Kleisin Rec8

The cohesin protein complex includes coiled-coil proteins with an SMC (Structural Maintenance of Chromosome) domain connected by an α-kleisin (hereafter named kleisin) subunit, and accessory proteins involved in loading/unloading of the complex onto/from chromatin. Cohesins play an essential role in sister chromatid cohesion and centromere organization for chromosome segregation during mitosis (Yatskevich et al., 2019). Cohesins have a major role in chromosome organization by mediating contacts between different chromosomal regions through potential different mechanisms, one of which is loop extrusion, as demonstrated by *in vitro* and *in vivo* studies in somatic cells (Fudenberg et al., 2016; Goloborodko et al., 2016; Davidson et al., 2019; Kim et al., 2019).

**FIGURE 2:**
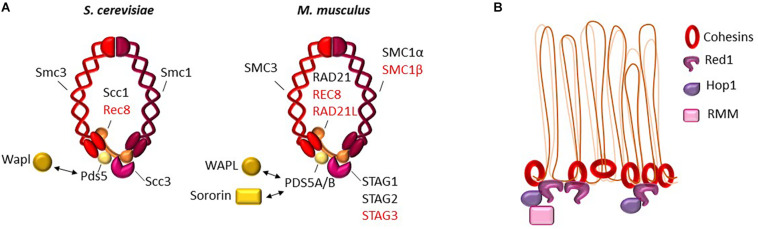
**(A)** The cohesin complex. Schematic presentation of the ring-like structure of the cohesin complex (red, pink, and orange), and the main regulators of its stability (yellow) in *S. cerevisiae* (left) and in mouse (right). Sororin-Pds5 interaction promotes stabilization of the cohesin complex and Wapl-Pds5 interaction promotes unloading of the cohesin complex. During meiosis in *S. cerevisiae*, the somatic α-kleisin subunit Scc1 (also called Rad21) is replaced by Rec8 (red). In mouse, several meiosis specific subunits exist (indicated in red), and differently combined lead to various cohesin complexes. Constitutive and meiotic orthologs of the various subunits are also listed in Table 1. **(B)** 2D localization of the cohesin complex and main partners in *S. cerevisiae. In S. cerevisiae*, cohesins and its main meiotic partners localize at axial elements at the basis of chromatin loops, where they contribute to loop-axis formation and sister chromatid cohesion. RMM is Rec114/Mei4/Mer2.

In *S. cerevisiae*, during vegetative growth, the cohesin complex is composed of four mains subunits, Smc1, Smc3, Rad21 (Scc1), and Scc3. During meiosis, Scc1 is expressed at very low levels, and a meiotic specific kleisin, Rec8, is expressed and ensures functions unique to meiotic cells (Figure 2). At the onset of meiosis, Rec8 is loaded on chromatin and forms the meiotic cohesin complex together with Smc1, Smc3, and Scc3. In addition to its role in sister chromatid cohesion at centromeres for ensuring chromosome segregation, Rec8 plays an essential role in meiotic chromosome structure and recombination (Klein et al., 1999). In the absence of Rec8, the axial structure of meiotic chromosomes is defective as observed by electron microscopy and Red1, one of the components of this structure, does not localize as linear structures (Klein et al., 1999). Genome-wide mapping showed that Rec8 localizes mostly around centromeres before meiotic S phase, but becomes enriched during meiotic prophase in intergenic regions of convergent genes, together with Red1 and Hop1, the structural proteins of axial elements (see below) (Kugou et al., 2009; Panizza et al., 2011; Sun et al., 2015). Rec8 localization coincides with regions of contacts, detected by HiC in yeast meiotic cells, that represent the bases of chromatin loops (Muller et al., 2018; Schalbetter et al., 2019).

Based on the interactions between Rec8 and Red1, it is thought that Rec8 recruits Red1/Hop1 to form chromosomal axis sites at the onset of meiotic prophase I (Sun et al., 2015; Figure 2). Similar interactions between cohesin and structural axis proteins have been observed in *Schizosaccharomyces pombe*, although it is Rec11, the meiotic-specific Scc3 subunit, and not Rec8 that interacts with the axial protein Rec10 (ortholog of Red1) (Sakuno and Watanabe, 2015).

The absence of Rec8 leads to defective axial structures of meiotic chromosomes and to changes in Red1 and Hop1 chromatin localization, but not to their loss (Panizza et al., 2011). In the absence of Rec8, Hop1/Red1 levels are decreased in some, but not all genomic regions. This indicates that Hop1 and Red1 can bind to chromatin in a cohesin-independent manner, but the determinants are not known. Interestingly, Red1 and Hop1 are less affected at small chromosomes in the absence of Rec8 (Panizza et al., 2011; Sun et al., 2015). Consistent with the view of Rec8 as a platform for Red1/Hop1 loading, in the absence of Red1, Rec8 chromatin loading is not affected (Sun et al., 2015).

### The Meiotic Axis Proteins Hop1 and Red1

The two chromosome axis proteins Hop1 and Red1 interact and form an evolutionarily conserved complex that can be recruited by cohesins. Hop1 and Red1 are required for homolog pairing and synapsis in *S. cerevisiae*, localize to meiotic chromosome axes (Hollingsworth and Byers, 1989; Rockmill and Roeder, 1990; Smith and Roeder, 1997), and are required for wild type level of meiotic DSBs and meiotic recombination (Mao-Draayer et al., 1996; Schwacha and Kleckner, 1997; Blat et al., 2002; Kim et al., 2010; Murakami et al., 2020). The formation of the Hop1 and Red1 complex is stimulated by the Mek1 kinase (delosSantos and Hollingsworth, 1999). These features are shared also by their orthologs in *S. pombe* (Hop1 and Rec10, respectively) (Lorenz et al., 2004; Kariyazono et al., 2019). Hop1 is a meiotic protein containing the HORMA domain. This domain, which was named after three proteins that harbor it (Hop1, Rev7, and Mad2), undergoes a regulated conformation change providing an alternative status for homo- and hetero-interactions (Rosenberg and Corbett, 2015). Structural analyses of the interaction between Hop1 and Red1 have identified an important feature: the interaction between a short domain called the closure motif, present on both Hop1 and Red1, and the HORMA domain. Red1 also oligomerizes to form protein filaments *in vitro* (West et al., 2018, 2019). The interaction features between Hop1 and Red1 are evolutionarily conserved in budding yeast, mammals, plants, and nematodes (Kim et al., 2014; West et al., 2019). Hop1 has DNA binding activity *in vitro* (Kironmai et al., 1998; Khan et al., 2012), but its direct implication *in vivo* remains to be demonstrated.

Hop1 and Red1 play a role in DSB formation by serving as a platform for the Mer2/Mei4/Rec114 DSB proteins (Panizza et al., 2011; Figure 2) that then promote Spo11 catalytic activity through direct or indirect interactions with Spo11 (Yadav and Claeys Bouuaert, 2021). This Hop1/Red1 function is mediated by the direct interaction between Hop1 and Mer2 in *S. cerevisiae* (Rousova et al., 2020), and between their orthologs Hop1 and Rec15 in *S. pombe* (Kariyazono et al., 2019). In the absence of Rec8, Hop1/Red1 and Rec114 still colocalize, but their positioning is altered (Panizza et al., 2011). DSB localization is also altered (Kugou et al., 2009), and DSBs are concentrated in regions enriched for Red1 and Hop1 (Sun et al., 2015). Hop1 or Red1 depletion does not have the same consequences. In the absence of Red1, Hop1 is not detected on chromatin, but DSB activity is still observed although at reduced levels. In the absence of Hop1, Red1 localization is not affected (Sun et al., 2015), but DSB activity is strongly reduced. Rec114 chromatin binding is strongly reduced in both cases (Panizza et al., 2011; Murakami et al., 2020).

Later during meiotic prophase I (i.e., after DSB formation), Rec8, Hop1 and Red1 play important roles in regulating the partner choice, homolog versus sister chromatid for DSB repair and by regulating DSB levels through the Tel1 kinase, and by switching off DSB activity upon Hop1/Red1 depletion from chromosomes concomitant with the formation of the synaptonemal complex (SC) that initiates at sites of DSB repair designed to mature as crossing overs (Keeney et al., 2014). Several structural changes take place to establish the tripartite structure of the SC composed of the two axes of each homolog and a central element (Zickler and Kleckner, 2015).

Overall, cohesin, Red1 and Hop1, by organizing chromosomes, have a key role in meiotic prophase. Two additional components of chromosome axis with direct and/or indirect role on meiotic recombination, DSB formation and/or repair are DNA Topoisomerase II (TopoII) (Klein et al., 1992; Zhang et al., 2014; Heldrich et al., 2020) and condensins (Yu and Koshland, 2003; Yu and Koshland, 2005; Hong et al., 2015).

## Localization of the Chromosome Axial Element in Mammals

In this section, we present the components of the mammalian axial element, involved in organizing meiotic chromosomes in early prophase I before the formation of the synaptonemal complex (Figure 1). These components include the cohesin complexes with somatic and meiotic-specific subunits (SMC1 α and SMC1β, SMC3, STAG3, RAD21, RAD21L, REC8) and their associated regulatory proteins (PDS5A, PDS5B, WAPL, and Sororin), the structural and regulatory proteins HORMAD1 and HORMAD2 and the axial element proteins SYCP2 and SYCP3 (Table 1).

**TABLE 1:** Evolutionary conservation of axis components.

	*S. cerevisiae*		*M. musculus*		*C. elegans*		*A. thaliana*		*D. melanogaster*	
	*Constitutive*	*Meiosis specific*	*Constitutive*	*Meiosis specific*	*Constitutive*	*Meiosis specific*	*Constitutive*	*Meiosis specific*	*Constitutive*	*Meiosis specific*
***Horma domain proteins***		Hop1		HORMAD1/2		HIM-3		ASY1/2		
						HTP-1/2/3				
***Axis associated proteins***		Red1		SYCP2				ASY3		
				SYCP3				ASY4		
***Cohesin complex subunits***										
*α-Kleisins*	Rad21 (Scc1)	Rec8	RAD21	REC8	SCC-1	REC8	RAD21.1/.2/.3 (SYN2/3/4)	REC8 (SYN1)	Rad21 (Scc1)	c(2)M
				RAD21L		COH-3/4				
*Coiled-coil domain proteins*	Smc1		SMC1α	SMC1β	HIM-1 (SMC-1)		SMC1		Smc1	
	Smc3		SMC3		SMC-3		SMC3		Smc3	
*Adapters, kleisin binding*	Scc3		STAG1/2	STAG3	SCC-3		SCC3		SA	
***Cohesin regulators***	Wapl		WAPL		WAPL-1		WAPl1/2		Wapl	
	Pds5		PDS5A/B		PDS-5 (Evl-14)		PDS5A/B/C/D/E		Pds5	
	Eco1 (Ctf7p)		ESCO1/2		F08F8.4		CTF7			
			Sororin							
***Cohesin loaders***	Scc2		NIPBL		PQN-85 (SCC-2)		SCC2		Nipped-B	
	Scc4		MAU2		MAU-2		SCC4		Mau2	

*The homologs of meiotic axis components in five different species (S. cerevisiae, M. musculus, C. elegans, A. thaliana, and D. melanogaster) are indicated, their expression is either constitutive or meiotic specific. Note that whether the constitutive components are also expressed in meiotic cells has not been always determined. The level of conservation varies: the subunits of the cohesin complexes, the cohesin regulators and loaders are relatively well conserved. The Horma domain allows the identification of homologs in the different species excepted for D. melanogaster. The axis associated proteins are poorly conserved at the amino-acid sequence level and were identified as homologs based on in vivo and in vitro data.*

### Cohesins

Like their mitotic counterparts and similarly to *S. cerevisiae*, mammalian meiotic cohesin complexes are composed of four core units that form a ring-like structure: two subunits of the SMC family, one α-kleisin, and one stromal antigen protein subunit (Table 1). In meiosis, there are three main cohesin complexes. All contain the two subunits SMC3 and SMC1β (Revenkova et al., 2001) and the accessory subunit STAG3 (or SA3), orthologous to *S. cerevisiae* Scc3 (Pezzi et al., 2000; Bayes et al., 2001; Prieto et al., 2004). Conversely, each of the three complexes contains a distinct kleisin subunit: RAD21 (Parra et al., 2004), RAD21L (Gutierrez-Caballero et al., 2011; Ishiguro et al., 2011; Lee and Hirano, 2011), or REC8 (Parisi et al., 1999; Eijpe et al., 2003; Lee et al., 2003). SMC1β (Revenkova et al., 2001; Eijpe et al., 2003), RAD21L (Herran et al., 2011; Ishiguro et al., 2011), REC8 and STAG3 are meiosis-specific proteins. In addition, the SMC1α subunit, present in the somatic cohesin complex, is also present in meiosis, leading to more complexity (Eijpe et al., 2003; Revenkova et al., 2004). In mice, deficiency for either of the meiotic-specific subunits leads to severe defects in meiotic prophase I, with variable phenotypes according to the subunit. Such defects are for example the impairment of sister chromatid cohesion, aberrant axis formation, defective recombination and homologous synapsis, and compromised telomere integrity, all of which ultimately lead to male and female sterility (Revenkova et al., 2004; Xu et al., 2005; Adelfalk et al., 2009; Herran et al., 2011; Biswas et al., 2013; Fukuda et al., 2014; Llano et al., 2014; Winters et al., 2014; Biswas et al., 2018).

#### The α-Kleisin Subunits of Cohesin

The first germ-cell specific cohesin complex that localizes on meiotic chromosomes contains REC8. In mouse male meiosis, where most of the observations were made, REC8 is detected early before the premeiotic S phase, when it starts to form foci, visible by immunostaining (Eijpe et al., 2003). Shortly afterward, in premeiotic S phase, the RAD21L-cohesin complex also starts forming foci all over the nucleus (Lee and Hirano, 2011; Ishiguro et al., 2014; Fujiwara et al., 2020). After premeiotic S phase, REC8 cohesin and RAD21L cohesin complexes show differences in localization on meiotic chromosomes. From the beginning of leptotene, REC8 cohesin complexes form foci that coincide with the staining of axial elements. On the other hand, RAD21L cohesin complexes are mainly detected around heterochromatin, where they form aggregates of bright foci. By mid-leptotene, RAD21L cohesin complexes change their pattern and form short axial structures (Ishiguro et al., 2011; Fukuda et al., 2014). Then, foci or short stretches of both REC8- and RAD21L-containing complexes progressively become almost continuous segments by zygotene (Eijpe et al., 2003; Herran et al., 2011; Ishiguro et al., 2011; Lee and Hirano, 2011; Fukuda et al., 2014). Subtle differences in localizations may also be due to distinct properties (sensitivity and specificity) of the antibodies used in such assays. At the beginning of pachytene, both complexes remain associated with the synapsed autosomal axis, and on the unsynapsed XY-bivalent (Ishiguro et al., 2011). REC8 co-localizes with the lateral elements of the SC, and RAD21L shows a more central localization in the SC, suggesting a distinct activity (Rong et al., 2016). The RAD21L signal starts to dissociate from the axis around mid-pachytene, partially producing self-assembled poly-complexes or aggregates. REC8 cohesin complexes remain strongly associated with synapsed and unsynapsed axes until diplotene, and then progressively disassemble to be detected as discontinuous foci along the chromosome arms and at centromeric regions (Ishiguro et al., 2011; Biswas et al., 2013; Fukuda et al., 2014). After prophase I, some residual RAD21L signal can be detected on chromosome arms (Herran et al., 2011; Ishiguro et al., 2011), but it is mostly restricted to centromeric regions and the unsynapsed sex chromosomes (Herran et al., 2011; Ishiguro et al., 2011; Lee and Hirano, 2011).

Through prophase I, the localization of RAD21L and REC8 on chromosomes is mostly mutually exclusive, suggesting that they may have distinct roles in axial element formation and sister chromatid cohesion (Ishiguro et al., 2011; Lee and Hirano, 2011; Vara et al., 2019). Interestingly, in zygotene, both complexes can be found in a symmetrical localization pattern between the two homologous unsynapsed regions of a given pair of chromosomes. This suggests the existence of intrinsic loading sites for cohesin-enriched domains featuring REC8 or RAD21L (Ishiguro et al., 2011; Lee and Hirano, 2011). However, a genome-wide chromatin immunoprecipitation (ChIP) study in pachytene cells, and thus the evaluation of the binding average of both proteins in a cell population, showed that most REC8 and RAD21L sites overlap and are located to active promoters (Vara et al., 2019).

#### Other Cohesin Complex Components

The somatic subunit SMC1α, present at premeiotic S-phase disappears at leptotene, and then reappears at late zygotene. At this stage, its phosphorylated form is detected as discontinuous stretches on synapsed chromosomes, including in the pseudo-autosomal region (PAR) of the sex chromosomes X and Y, and its non-phosphorylated form marks the chromatin loops of the XY-bivalent. By the end of diplotene, both phosphorylated and non-phosphorylated SMC1α disappear completely (Eijpe et al., 2000, 2003; Biswas et al., 2013; Hopkins et al., 2014). After premeiotic S phase, SMC1β largely replaces the somatic subunit SMC1α (Revenkova et al., 2001). From leptotene, SMC1β localizes along the forming axial elements, rather uniformly along unsynapsed and synapsed autosomes, as well as on the unsynapsed XY-bivalent at pachytene. From leptotene to early pachytene, SMC1β is detected also at telomeres (Adelfalk et al., 2009). At the beginning of diplotene, SMC1β initially stays on desynapsed chromosomes, but also starts to accumulate at centromeric regions. Then, it progressively dissociates from the chromosome arms, and remains only at the centromeric regions until the metaphase II–anaphase II transition (Revenkova et al., 2001). In agreement with their distinct spatiotemporal localization, SMC1α and SMC1β do not interact within the same complex, but they both associate with SMC3, as indicated by immunoprecipitation (Ishiguro et al., 2011; Lee and Hirano, 2011). SMC3 is present at all stages of meiosis, and its staining pattern is compatible with both isoforms (Eijpe et al., 2003). Thus, during the axial element formation in early prophase I, the vast majority of meiotic cohesin complexes contain SMC3 and the meiosis-specific SMC1β isoform.

The meiosis-specific stromalin subunit STAG3 appears first at preleptotene when it localizes to telomeres and chromocenters (Shibuya et al., 2014). From leptotene, STAG3 expression completely overlaps with that of SMC1β in time and space (Prieto et al., 2001). This suggests that at this time point of axial element formation, cohesin complexes contain SMC1β, SMC3, and STAG3.

The third cohesin complex present during meiosis contains the somatic RAD21 subunit. Some studies reported RAD21 presence on chromosome axes and centromeres from leptotene to diplotene (Xu et al., 2004; Herran et al., 2011; Llano et al., 2012), whereas others detected RAD21 only from late pachytene onward (Ishiguro et al., 2011; Lee and Hirano, 2011). At pachytene and diplotene, RAD21 rarely colocalizes with RAD21L or REC8 (Ishiguro et al., 2011; Lee and Hirano, 2011; Fukuda et al., 2014). This could indicate that RAD21 either binds to distinct sites, or replaces the meiosis-specific RAD21L and REC8 cohesin complexes when they dissociate from the chromosome arms by the end of pachytene and diplotene, respectively. Later, from diakinesis until anaphase II, RAD21 is only detected at inner centromeres (Parra et al., 2003, 2004; Xu et al., 2004).

The stability and persistence time of cohesin complexes on chromosomes are regulated by pro- and anti-cohesion factors (e.g., Sororin, ESCO1, ESCO2, WAPL, and PDS5) and several posttranslational modifications (Schmitz et al., 2007; Shintomi and Hirano, 2009; Nasmyth, 2011; Losada, 2014; McNicoll et al., 2020). In somatic cells, cohesins can be actively released by two pathways: the Separase and the WAPL-PDS5 pathways (Nasmyth et al., 2000; Peters and Nishiyama, 2012). Separase catalyzes the proteolytic cleavage of the kleisin subunit of cohesins during metaphase–anaphase transition. On the other hand, WAPL promotes cohesin unloading during mitotic prophase, and to some extent during interphase, through an antagonistic mechanism that involves the competition with Sororin for binding to PDS5 (Gandhi et al., 2006; Kueng et al., 2006; Haarhuis et al., 2013; Tedeschi et al., 2013; Ouyang et al., 2016). Thus, Sororin-PDS5 promote the loading and stabilization of cohesins, and WAPL-PDS5 mediate their unloading.

**FIGURE 3:**
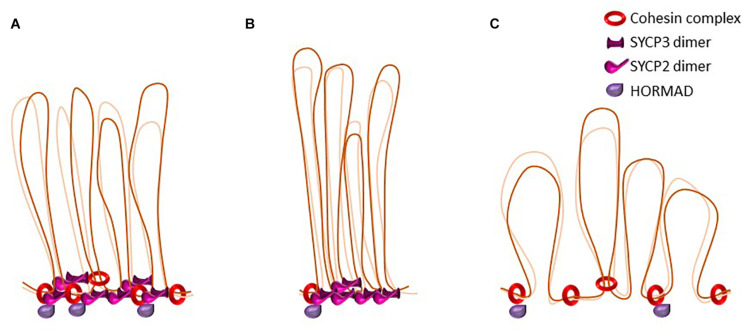
The dynamics of loop-axis organization. Schematic representation of axial elements in the mouse. Cohesins form the axial core onto which other axial element proteins such HORMADs, SYCP2, and SYCP3 bind. Some cohesin complexes are involved in sister cohesion, others might be involved in the formation of the loop axis-structure. SYCP3 and SYCP2 form an antiparallel heterotetramer. **(A)** representation of wild type axial elements. **(B)** Representation of loop-axis structure in mouse mutants for the cohesin subunit SMC1β. Axes are twofold shorter and loops appear longer and less homogenous in size. In this mutant, HORMAD is able to load on axes. **(C)** Representation of loop-axis structure in mice deficient for SYCP3. Cohesin axis core is formed but axes are almost twofold longer and loops appear shorter, irregular, and denser. SYCP2 is not detected on axes in the absence of SYCP3.

In prophase I, pro-and anti-cohesin factors have similar functions as in somatic cells, but they face additional challenges due to the different constraints that are related to the presence of other axis associated proteins such as HORMADs, SYCP2, and SYCP3 (see below, also Figure 3A). At that stage, the unloading of cohesin complexes is regulated by WAPL and the two orthologs, PDS5A and PDS5B, both strongly expressed in mouse testes and ovaries (Kuroda et al., 2005; Losada et al., 2005; Zhang et al., 2008; Fukuda et al., 2010). WAPL is detected at lateral axial elements of zygotene and pachytene spermatocytes, and colocalizes with SYCP2 (Kuroda et al., 2005; Zhang et al., 2008). Similarly, PDS5A is detected on axial and lateral elements from zygotene until early pachytene. By mid-pachytene PDS5A disperses on the chromatin, with no staining detected on lateral axial elements. Later, at metaphase I and II, PDS5A reappears at centromeres (Viera et al., 2020). PDS5B has a partly different localization pattern, it is detected earlier (early leptotene) and remains longer (late pachytene) at axial elements where it colocalizes with REC8, and partially with SYCP3 (Viera et al., 2020). Co-immunoprecipitation assays corroborate this finding, showing that PDS5B interacts with the axis-related proteins SMC1β, SYCP2, and HORMAD1 (Fukuda et al., 2010). PDS5B also colocalizes with telomeres in all stages of prophase I (Viera et al., 2020). Conditional depletion of either PDS5A or PDS5B does not alter progression through prophase I. However, the simultaneous depletion of both proteins leads to severe defects in axial element formation (see below) and telomere integrity. This indicates that albeit their different localization pattern, PDS5A and PDS5B have redundant functions (Viera et al., 2020).

Rather surprisingly, the WAPL antagonist Sororin localizes only in the central region of the synaptonemal complex, whereas the other subunits of the cohesin complex do not localize in this region, and its presence on meiotic chromosome axes correlates with the appearance of the central element protein SYCP1. This suggests that in meiosis, Sororin might have different functions from what described in mitosis (Gomez et al., 2016; Jordan et al., 2017). ESCO1 role in meiosis has not been investigated yet, but ESCO2 and acetylated SMC3 are mostly detected upon synapsis formation in zygotene (McNicoll et al., 2020). Both proteins remain associated with the chromosome axes until desynapsis in diplonema, suggesting that acetylated SMC3-stabilized cohesion is required during prophase I, and likely during synaptonemal complex formation or maintenance. Germ cell-specific conditional ablation of *Esco2*, resulting in ESCO2 hypomorphism in spermatocytes, leads to a delay in synaptonemal complex formation, a slight defect of autosome synapsis, and a clear defect in sister chromatid cohesion of unsynapsed sex chromosomes that appears transiently in pachynema, adjacent to the XY PAR. This suggests a role of ESCO2 in sister chromatid cohesion of unsynapsed regions in addition to supporting autosomal synaptonemal complex synapsis (McNicoll et al., 2020).

The role of ESPL1 Separase has been investigated in mouse oocytes only, and specific depletion of ESPL1 in metaphase arrested oocytes shows that ESPL1 promotes the release of REC8 from chromosome arms and allows chiasma resolution (Kudo et al., 2006; Tachibana-Konwalski et al., 2010). In oocytes, the REC8 cohesin complex is mostly resistant to WAPL-mediated release. This is reminiscent of what observed in *Caenorhabditis elegans*, where WAPL releases COH-3/4 but not REC8 cohesin complexes, and COH-3/4 is functionally linked to RAD21L (Severson and Meyer, 2014; Silva et al., 2020).

### The HORMA Domain Proteins HORMAD1 and HORMAD2

HORMA domain-containing proteins are conserved in yeast (Hop1), plants (ASY1), nematodes (HTP-1–3 and HIM-3) and in mammals (Table 1). *Hormad1* and *Hormad2* were discovered as genes that are specifically upregulated in female and male mouse gonads during prophase I (Wojtasz et al., 2009; Fukuda et al., 2010; Shin et al., 2010). Mouse HORMAD1 and HORMAD2 are closely related, and both have human homologs. Their HORMA domains are highly conserved; the HORMA domain of mouse HORMAD1 shares 28 and 89% of amino acids with the HORMA domain of *S. cerevisiae* Hop1 and human HORMAD1, respectively. Mouse HORMAD1 is a 44kDa protein; a slightly shorter isoform is weakly expressed and lacks the nuclear localization signal at its C-terminus (Fukuda et al., 2010). HORMAD2 is a 35 kDa protein with no other documented isoforms in the mouse. HORMAD1 and HORMAD2 show slightly different dynamics during prophase I (see below). In mouse, deficiency of HORMAD1 leads to sterility in male and female due to defects in recombination and in homologous synapsis, leading to an impairment of chromosome segregation. This indicates an important role of this protein in early prophase I events (Shin et al., 2010; Daniel et al., 2011). HORMAD2 deficiency leads to sterility in males only, likely due to a role of this protein in the surveillance of asynapsis upon synaptonemal complex formation (Kogo et al., 2012; Wojtasz et al., 2012).

*In vivo*, immunofluorescence and proximity ligation assays demonstrated that HORMAD1 appears as distinct foci at preleptotene, when it mostly co-localizes with REC8 and RAD21L cohesins. Super-resolution microscopy showed that RAD21L tends to colocalize more frequently with HORMAD1 than REC8 (Fujiwara et al., 2020). At leptotene, both HORMAD proteins form short stretches that overlap with RAD21L, REC8 and the axial element proteins SYCP3 and SYCP2 (see below) (Wojtasz et al., 2009; Shin et al., 2010; Fujiwara et al., 2020). At this stage, the chromosomal localization of HORMAD1 is slightly reduced in the absence of SYCP2, suggesting that this protein might be involved in HORMAD1 stabilization on chromosomes (Fujiwara et al., 2020).

During zygotene, HORMAD1 and HORMAD2 staining is stronger on unsynapsed than synapsed axes, and the signal intensity is weaker close to centromeres. Synapsed regions show some residual HORMAD signal the intensity of which is anti-correlated with that of synaptonemal complex proteins, such as SYCP1, indicating HORMAD1 and HORMAD2 depletion upon synaptonemal complex formation. The depletion of HORMADs from synapsed regions is independent of meiotic recombination but depends on TRIP13, an ortholog of *S. cerevisiae* Pch2, and on the formation of the synaptonemal complex (Wojtasz et al., 2009; Dereli et al., 2021). In *S. cerevisiae*, Pch2 is also involved in Hop1 depletion from synapsed axes (Borner et al., 2008). In pachytene, the signal for both HORMAD proteins is strongest on the unsynapsed XY-bivalent (in males), but some punctuate signal remains along synapsed chromosomes. At this stage, HORMAD1 is present as two distinct forms: phosphorylated and non-phosphorylated (Wojtasz et al., 2009; Shin et al., 2010). Although HORMAD1 and HORMAD2 mostly overlap until pachytene, they show slightly distinct distribution patterns. In zygotene, HORMAD2 signal spreads further in the synapsed regions than HORMAD1, indicating that HORMAD1 is depleted faster from the forming synaptonemal complexes than HORMAD2. During pachytene, HORMAD1 is clearly detectable on centromeres of synapsed chromosomes, whereas HORMAD2 shows only a very faint signal in these regions. In spermatocytes during diplotene, HORMAD1 and HORMAD2 show distinct patterns on the axes of desynapsing autosomal chromosomes. HORMAD1 signal increases, whereas HORMAD2 remains restricted to the unsynapsed XY-bivalent. Conversely, in diplotene oocytes, both are detected on desynapsed axes. During diakinesis, only HORMAD1 strongly accumulates at inner centromeres and remains between sister-kinetochores until interkinesis, perfectly overlapping with the SYCP3 signal (see below) (Wojtasz et al., 2009; Fukuda et al., 2010).

### The Axial Proteins SYCP2 and SYCP3

Synaptonemal complex protein 3 (SYCP3, also called SCP3 and Cor1) was first described as a component of rat and hamster axial elements (Heyting et al., 1985, 1987; Dobson et al., 1994; Lammers et al., 1995). No ortholog has been identified in yeasts, but orthologs were found in many metazoans, including in early branching lineages, such as *Cnidaria* (Fraune et al., 2012). In almost all studied species, the *Sycp3* gene encodes a single protein product, except in the mouse and rat, where it encodes an additional longer isoform, with an N-terminal extension. It is not clear whether this isoform has additional properties (Alsheimer et al., 2010). Mammalian SYCP3 is a fibrillar 30 kDa molecule that is composed of a central α-helical domain that is flanked by non-helical N- and C-termini, and that interacts with double-stranded DNA *via* its N-terminal DNA binding domain (Syrjanen et al., 2014). A crystallographic analysis showed that the N-terminal regions are located at both ends of a SYCP3-tetramer, composed of a helical core, which folds in an elongated rod-like structure. Single molecule fluorescent microscopy provided *in vitro* evidence that this structural feature allows SYCP3 to hold distant DNA regions together via a non-sequence specific bridging reaction (Syrjanen et al., 2017). However, *in vivo*, SYCP3 might show some sequence specificity, because ChIP and DNA sequencing in macaque, mouse and rat demonstrated that it associates with a specific subfamily of active short-interspersed elements (SINE) (i.e., AluY and B1). SYCP3 might use these sequences as anchoring points, while repressing their retrotransposition activity (Hernandez-Hernandez et al., 2008; Johnson et al., 2013). In female mice, deficiency of SYCP3 promotes aneuploidy due to segregation defects in oocytes, leading to non-viable offspring (Yuan et al., 2002; Kouznetsova et al., 2005; Wang and Hoog, 2006). In male, the absence of SYCP3 compromises the maintenance of the integrity of axial elements and the efficiency of the repair of meiotic double strand breaks, leading to a prophase I arrest (Yuan et al., 2000; Pelttari et al., 2001).

*In vitro* assays showed that SYCP3 forms tetramers, bind DNA and can self-assemble into regular superstructures that are reminiscent of the structure of axial elements (Syrjanen et al., 2014; West et al., 2019). Those studies further indicated that this self-assembly property does not depend on the presence of DNA. However the SYCP3 fiber structure can interact with DNA through the N-terminal tail (Syrjanen et al., 2014; Bollschweiler et al., 2019). It was proposed that the structural properties of SYCP3 fibers are compatible with those of a liquid crystal (Rog et al., 2017), and that SYCP3 might form a protein layer, coating the surface of the existing chromosome structure established by cohesins (Bollschweiler et al., 2019). SYCP3 physical properties might also explain the evolutionarily conserved density of chromatin loops (∼20 loops per μm) on meiotic chromosomes (Kleckner, 2006), because molecular modeling predicts one loop for every two repeating units of the SYCP3 fiber (Syrjanen et al., 2014). It is unclear what determines the length of the loops and in turn the length of the chromosome axis. Cohesins might be involved in regulating loop length, and also in axis length together with other axis components (see below section “Axis Formation and Axis-Loop Organization”).

*In vivo*, it is likely that SYCP3 assembles on the pre-existing chromosome axis, determined to a large extent by cohesins (see below). Immunostaining of spermatocyte spreads showed that SYCP3 first appears at preleptotene/early leptotene, where it forms small stretches of axial-like structures that colocalize with REC8 and RAD21L cohesins, HORMADs and SYCP2 (see below). The association of SYCP3 with axial elements might be stabilized by SYCP2, because in its absence SYCP3 signal is reduced (Fujiwara et al., 2020). At this early stage, most centromere regions also co-localize with SYCP3 (Bisig et al., 2012). Like cohesins, SYCP3 then forms increasingly longer stretches of axial structures that become progressively more continuous and form the lateral elements of the synaptonemal complex in pachytene. Like all axial element components, SYCP3 localizes on unsynapsed and synapsed chromosomes and the XY-bivalent. Super-resolution imagining of SYCP3 staining in mouse spermatocytes showed that in pachytene, the lateral elements form a helicoidal structure around the central element of the synaptonemal complex (Schucker et al., 2015). In late diplotene, SYCP3 starts to dissociate from the chromosome arms and accumulates during diakinesis at the inner domain of centromeres. At metaphase I and interkinesis, SYCP3 is completely lost from the chromosome arms, and is detected as a bar-like structure between sister kinetochores. In telophase I, when sister kinetochores are separated, SYCP3 is released from kinetochores (Dobson et al., 1994; Parra et al., 2004, 2006, 2009; Bisig et al., 2012).

Synaptonemal complex protein 2 (SYCP2, also called SCP2) was first identified as a component of rat axial elements. Its sequence shows limited homology over a short region with the yeast protein Red1 (Offenberg et al., 1998). Biochemical and structural studies found similarities between HORMAD1-SYCP2, Hop1-Red1 in yeast and ASY1-ASY3 in *Arabidopsis thaliana*, thus indicating the evolutionary conservation of this pathway of axis assembly (Table 1; West et al., 2019). Mouse SYCP2 is a 172 kDa protein harboring different potential DNA binding motifs and several distinct domains. The crystal structure of the ordered N-terminal domain revealed two separate subdomains, ARLD and SLD. The ARLD domain might function as a protein-interacting platform, because it can associate with various proteins, such as the two centromere-associated proteins CENP J and F. The SLD domain structurally resembles the Spt16M subunit of the FACT complex, a chaperone involved in the assembly and disassembly of histones H2A and H2B. This domain might be implicated in SYCP2 binding to chromatin (Feng et al., 2017). The N-terminal region of SYCP2 is followed by an extended disordered region. A short region, directly following the ordered region of the N-terminal domain shows homology with HORMAD1 and HORMAD2 closure motif. This putative closure motif, the position of which is equivalent to the closure motif in Red1, interacts with the HORMA domain of HORMAD2 and competes with the HORMAD2 closure motif to bind to the HORMA domain of HORDMAD2. It is predicted (not demonstrated yet) that the SYCP2 closure motif also binds to HORMAD1 (West et al., 2019). The C-terminal of SYCP2 contains a coiled-coil domain. Unlike Red1 that self-associates through its coiled-coil domain, SYCP2 interacts with the coiled-coil domain of SYCP3 (Tarsounas et al., 1997; Yang et al., 2006; Winkel et al., 2009; West et al., 2019). Although SYCP3 can exist as a homo-tetramer (Syrjanen et al., 2014; West et al., 2019), antiparallel SYCP2:SYCP3-heterotetramer formation appears to be the preferred state when both proteins are present (West et al., 2019).

*In vivo*, SYCP2 first appears at meiotic axial elements in early leptotene (Schalk et al., 1998). Until metaphase I, SYCP2 co-localizes with SYCP3 (Kouznetsova et al., 2005; Shin et al., 2010). High-resolution stochastic optical reconstruction microscopy revealed that the C-terminal, coiled-coil domain of SYCP2 preferentially co-localizes with SYCP3, compared with the N-terminus, as predicted by interaction data (Xu et al., 2019). SYCP2 localization on chromosomes depends on SYCP3 and cohesins (see below) (Pelttari et al., 2001; Fujiwara et al., 2020). In mouse, absence of SYCP2 leads to male sterility and female subfertility, due to their incapacity of forming fully functional axial elements (Yang et al., 2006; Fujiwara et al., 2020).

## Functional Roles of the Meiotic Axis Organization in Early Meiotic Prophase I

### Axis Components and the Control of Sister Chromatid Cohesion

**FIGURE 4:**
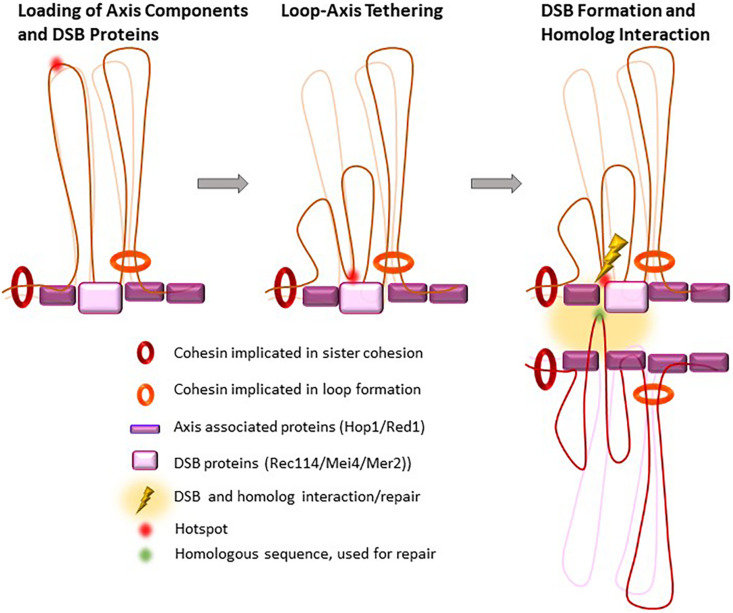
Proposed loop re-organization at leptonema upon and after DSB formation. In early prophase I, meiotic hotspots localize in loops and the DSB formation proteins (DSB pre-recombinosome) localize on the axis. Before DSB formation, hotspots contact the DSB pre-recombinosome, reorganizing the loops that contain hotspots where DSB will form.

The general principles of the establishment and maintenance of sister chromatin cohesion (SCC) also apply to the meiotic stages. The complexity of SCC establishment and maintenance at chromosome arms during early prophase I results from the presence of different kleisin subunits that in agreement with their differential spatiotemporal localization, appear to have different properties. FISH analysis in spermatocytes that lack REC8 and RAD21L (the two meiotic kleisins) shows proper SCC at prophase I entry, suggesting that the somatic RAD21 cohesin complexes are sufficient to establish SCC during premeiotic S phase (Llano et al., 2012). It is likely that RAD21L has no or only a minor role in SCC along chromosome arms, because in its absence their cohesion is established and maintained through prophase I (Herran et al., 2011; Ishiguro et al., 2014). On the other hand, REC8 cohesin complexes play an important role in SCC at chromosome arms early in prophase I (Figure 4). In REC8 absence, cohesion is established but sister chromatids partially lose their tight association and split, although RAD21L cohesin complexes can load onto chromosome axes (Bannister et al., 2004; Xu et al., 2005; Agostinho et al., 2016). In STAG3-deficient mice, in which REC8 levels are reduced, it appears that REC8 density along chromosome arms is crucial for SCC maintenance. If the spacing of REC8 foci is larger than 15% of the total chromosome length, the tight association is lost (Agostinho et al., 2016). SMC1β absence also leads to the local loss of the tight association between sister chromatids on a proportion of chromosomes from early prophase. The relatively milder SCC phenotype, compared with what observed in the absence of REC8, may be due to the presence of SMC1α that can compensate for SMC1β role in SCC at chromosome arms (Revenkova et al., 2004; Biswas et al., 2013; Agostinho et al., 2016). Absence or reduction of STAG3 partially phenocopies the loss of REC8 because the amount of REC8 cohesin complexes is strongly reduced in these mice (Caburet et al., 2014; Fukuda et al., 2014; Hopkins et al., 2014; Llano et al., 2014; Winters et al., 2014). However, unlike REC8 mutants (Bannister et al., 2004), the cohesion of centromeres and telomeres is partially lost, showing the important role of STAG3 in SCC maintenance, which is not compensated by the somatic STAG1 and STAG2 isoforms even though they are detected in meiosis (Fukuda et al., 2014; Llano et al., 2014; Winters et al., 2014).

Interestingly, all cohesin mutants that lead to loss of SCC at chromosome arms (SMC1β, REC8 and STAG3) present loading of synaptonemal complex proteins (SYCP1, SYCE1, SYCE2 and TEX12) between sister chromatids instead of homologs (Bannister et al., 2004; Xu et al., 2005; Agostinho et al., 2016; Biswas et al., 2016). It is not clear whether the loss of the tight association between sister chromatids allows the formation of inter-sister synaptonemal complexes, or whether the binding of REC8 cohesin complexes prevents the binding of synaptonemal complex proteins to specific sites on axial elements (Bannister et al., 2004; Xu et al., 2005; Caburet et al., 2014; Fukuda et al., 2014; Hopkins et al., 2014; Llano et al., 2014). The role of RAD21L is not known. RAD21L cohesin complexes do not mediate SCC, but may promote contacts and loop organization on single chromatids or between recombinant chromatids. This duality of function between REC8 and RAD21L is observed also for the *Drosophila melanogaster* Rad21 and C(2)M proteins (Cahoon and Hawley, 2016; Gyuricza et al., 2016) and in *C. elegans* where REC8 and COH-3, COH-4 (RAD21L orthologs) (Severson et al., 2009) show distinct localizations on chromosome axes (Kohler et al., 2017; Woglar et al., 2020). However, this separation of tasks between REC8 and RAD21L homologs is not a general rule as some species have either REC8 only (*S. cerevisiae*; *A. thaliana*), as RAD21L is only found in deuterostomia, or RAD21L only (birds), as meiotic-specific kleisin (Gutierrez-Caballero et al., 2011).

### Axis Formation and Loop-Axis Organization

#### Contribution of Axis Components

Extensive studies in single and double mutant mice demonstrated that cohesins are not only the first building blocks of axial elements, by forming an axial chromosome core upon which the remaining axial element proteins can build, but they also determine their structure, and specifically their length. Their role in axis formation seems not to be linked to their function in SCC, because the axis can form normally in a *Coprinus cinereus* mutant that lacks sister chromatids or in mutants where the premeiotic S-phase does not occur (Merino et al., 2000; Blitzblau et al., 2012). The most striking phenotype, which shows the importance of cohesins in axis formation, is observed in STAG3-deficient mice, where the axis does not form, and only dot-like structures remain that might just contain SYCP3 aggregates (Ishiguro et al., 2014; Winters et al., 2014). The presence of cohesin subunits on axis in a distinct *Stag3* mutant background may be due to residual level of STAG3 and/or expression of STAG1/2 (Caburet et al., 2014; Fukuda et al., 2014; Hopkins et al., 2014; Llano et al., 2014).

Absence of SMC1β also dramatically alters the axis structure with almost a twofold reduction of its length in *Smc1β*–/– mice compared with wild type mice (Revenkova et al., 2004; Biswas et al., 2016). The different kleisin proteins show distinct functions during axis formation. Concomitant absence of REC8 and RAD21L leads to a similar phenotype as in STAG3-deficient mice, suggesting that somatic RAD21 contributes only little, or not at all, to the formation of the chromosomal axial core (Llano et al., 2012; Biswas et al., 2016). However, absence of REC8 or RAD21L alone only partially affects axis length, with a length reduction of about one third compared with wild type (Biswas et al., 2016). Genetic studies using mice that lack SMC1β and one of the two kleisins brought more information on their relative involvement. The combined loss of SMC1β and REC8 reduces axis length to one third of the wild type length. Conversely, the simultaneous loss of SMC1β and RAD21L is almost as dramatic as the loss of STAG3, with a seven-fold reduction of the axis length compared with wild type. These data show that axis formation is mainly supported by the meiotic cohesin components SMC1α/RAD21L, SMC1β/REC8 and to a lower extent by SMC1α/REC8 (Biswas et al., 2016). Interestingly, axis length can also be impacted by the dynamic behavior of cohesin complexes. Indeed, the concomitant depletion of the cohesin regulators PDS5A and PDS5B leads to a severe shortening of axial elements, despite the presence of all tested cohesin subunits in those spermatocytes (Viera et al., 2020). This role of Pds5 in controlling axis length as also been observed in *S. cerevisiae* (Jin et al., 2009; Song et al., 2021) and even in the absence of the sister chromatid (Hong et al., 2019). Although this may indicate an increase in loop lengths, this may result from the consequences of the absence of Pds5 on loop expansion and on sister chromatid cohesion as clearly observed by HiC in yeast vegetative cells (Dauban et al., 2020).

The severe defects of axis formation observed in mutants for cohesins and their regulators, illustrate the importance for forming a functional cohesin core that provides the structural basis for the loading of other proteins such as HORMADs, SYCP2, and SYCP3 to complete axial element formation. Yet, not all cohesin complexes present in prophase I have equivalent roles for the recruitment of other axis-associated proteins. REC8 deficiency, for example only slightly affects HORMAD1 foci formation, as shown by immunostaining. Conversely, the number of preleptotene and early leptotene HORMAD1 foci is drastically reduced in RAD21L mutants, suggesting, at least at early stages, the involvement of RAD21L in HORMAD1 loading to chromosomes (Fujiwara et al., 2020). The lack of a definite DNA binding domain in both HORMAD proteins and the fact that HORMAD1 co-immunoprecipitates with all meiotic cohesin subunits, corroborate this possibility (Fujiwara et al., 2020). It has also been suggested, that HORMAD1 and RAD21L stabilize each other for their mutual loading on chromatin, because RAD21L axis localization also is slightly reduced in the absence of HORMAD1, without any obvious effect on cohesin axial core formation or SYCP3 or SYCP2 loading (Shin et al., 2010; Daniel et al., 2011; Fujiwara et al., 2020). Thus, HORMAD1 loading depends on cohesin components and might be coordinated with RAD21L loading or stabilization. Additionally, even though HORMAD1 is loaded independently of axial element proteins, as shown by genetic studies in mice lacking SYCP2 or SYCP3 (Fukuda et al., 2010; Fujiwara et al., 2020), those proteins may play a role in stabilizing HORMAD1 interaction with the axial core because cohesin-HORMAD1 co-localization is reduced in SYCP2-deficient mice (Fujiwara et al., 2020).

SYCP2 and SYCP3 are not essential for the formation of the cohesin core (Yuan et al., 2000, 2002; Pelttari et al., 2001; Fujiwara et al., 2020), but have an important role in determining its compaction and thereby the size of axial elements. In SYCP3-deficient oocytes, the residual axis is twice as long as in wild type cells (Yuan et al., 2002; Novak et al., 2008). The respective contribution of each protein is difficult to address because the loading of one protein on the axial core depends on the other (Pelttari et al., 2001; Yang et al., 2006; Fujiwara et al., 2020). This default in axis compaction can be partly rescued by the simultaneous absence of SMC1β and SYCP3, leading to the hypothesis that the interplay between the compaction exerted by axial element proteins and the restriction of this compaction mediated by cohesin complexes determines the final length of axial elements in meiosis (Novak et al., 2008).

#### The Loop-Axis Configuration

The loop-axis organization of prophase I chromosomes is a very fascinating aspect of meiosis. The first description of the loop-axis organization in the mouse was based on light microscopy observations in which pachytene chromosomes were described as axes from which emanate fuzzy lateral projections, interpreted as DNA loops (Monesi, 1965), as previously suggested in rat and insect spermatocytes (Sotelo and Trujillo-Cenoz, 1960). The lamp brush-like loop-axis shape of meiotic chromosomes in the mouse was later confirmed by electron microscopy (Kierszenbaum and Tres, 1974). The general nature of the loop-axis setup of meiotic chromosomes is extremely conserved in different species (Zickler and Kleckner, 1999). However, the fine-scale loop-axis organization may vary greatly, even within species. An example of this variation can be observed in male and female mammals. Immunofluorescence studies and electron microscopy serial sections (von Wettstein et al., 1984) in mouse and human, have shown that axes are in average twofold longer in female than in male gametocytes with variations between chromosomes (Tease and Hulten, 2004; Gruhn et al., 2013; Wang et al., 2017). The mouse PAR region on the XY chromosomes further illustrates variability between chromosomes. In early prophase, the PAR axis is longer compared with that of autosomal regions of similar size and then shortens as prophase I progresses. In both examples, staining of chromatin loops by immuno-FISH shows that longer axes (female and early PAR) feature more compact loops, that appear closer to the axis, whereas shorter axes (male and late PAR) feature more decompacted loops, that appear further away from the axis (Kauppi et al., 2011; Acquaviva et al., 2020). Similar observations were made in mutant mice where axes are shorter (Cohesin and PDS5, see above) or longer (SYCP3, see above) (Novak et al., 2008; Viera et al., 2020; Figure 3). Taken together, these observations led to the suggestion that in mouse and human, axis-lengthening leads to loop shortening and *vice-versa*, as it was proposed in other organisms (Zickler and Kleckner, 1999; Tease and Hulten, 2004; Kleckner, 2006). However, it is difficult to infer loop length from Immuno-FISH experiments on chromosome spreads. Loops that appear shorter, might contain the same amount of chromatin as loops that appear longer, but simply stretch out laterally due to a longer and less compact axis. Ideally, one would need to measure the amount of DNA present within loops. In addition, a change in axis length is expected to be associated to a change in loop length, only if one assumes that the density of loops per unit length is constant.

Loop organization has been analyzed by direct molecular approaches in particular by chromosome conformation capture (HiC). In *S. cerevisiae* HiC experiments showed that pachytene chromosomes feature a punctuate contact pattern in which contact points partially overlap with Rec8 binding sites (Muller et al., 2018; Schalbetter et al., 2019). This contact pattern is lost in the absence of Rec8 (Schalbetter et al., 2019), suggesting that cohesin is responsible for shaping the loop-axis conformation of prophase I chromosomes in yeast. *In silico* simulation experiments, in which chromatin is modeled as a polymer, best fit with the experimental data when Rec8 is localized at the basis of chromatin loops that are progressively extruded, until stopped by another extruder or an unknown barrier, for example a large protein complex, such as the transcription machinery. The best fitting model suggests that in a given cell, only few Rec8 sites, not necessarily adjacent, are occupied, leading to the heterogeneous loop size distribution observed along chromosomes. In this scenario, loop distribution is expected to be mostly stochastic, and thus, to vary from cell to cell, and possibly between sister chromatids and homologs. In these experiments, the loop size was estimated to be in the range of 10–50 kb, with a mean value of about 26 kb (Muller et al., 2018; Schalbetter et al., 2019). This estimation is consistent with the loop size of 20kb proposed by electron microscopy experiments (Moens and Pearlman, 1988).

In the mouse, HiC experiments using purified zygotene and pachytene spermatocytes show contact probability patterns that are compatible with chromosome individualization, and that are similar to what observed in mitotic cells, namely a pattern of a linear compressed array of consecutive loops along a scaffold axis (Gibcus et al., 2018). The establishment of the loop-axis configuration in the mouse is accompanied by a progressive loss of contacts of topologically associated domains (TADs), with similarity to mitosis. TAD boundaries are preferentially occupied by CTCF that can provide anchors for cohesin-mediated loop extrusion (Bonev and Cavalli, 2016). Intriguingly, unlike in mitosis where CTCF is largely evicted from chromosomes, CTCF remains bound to TAD boundaries in pachytene chromosomes (Oomen et al., 2019; Vara et al., 2019; Luo et al., 2020). Another feature lost in mitosis, but conserved in prophase I chromosomes, is the maintenance of a substantial amount of refined compartmentalization of large-scale, gene-dense *versus* gene-poor compartments, also called A and B compartments. These compartments are maintained in meiotic chromosomes possibly due to active transcription (Alavattam et al., 2019; Patel et al., 2019; Vara et al., 2019; Wang et al., 2019; Luo et al., 2020). Unlike in *S. cerevisiae*, in the mouse and monkey there is no evidence of distinct loop-basis interaction sites. This suggests that loops are mainly anchored randomly along axial elements, yielding only very low-frequency contact sites, which remain undetectable at the sensitivity of the experiments performed until now (Alavattam et al., 2019; Patel et al., 2019; Vara et al., 2019; Wang et al., 2019; Luo et al., 2020). If distinct loop interaction sites exist in the mouse, CTCF-bound sites, which colocalize with about half of all REC8 and RAD21L binding sites found in pachytene cells, would be good candidate regions (Vara et al., 2019; Luo et al., 2020).

*In silico* modeling using HiC data estimated the mean loop size of mouse zygotene and pachytene chromosomes to be between 0.8 and 2 Mbps (Alavattam et al., 2019; Patel et al., 2019). This value may, however, mask variability that is detected when separating A and B compartments and revealing shorter predicted loop sizes in A compartments (Jin et al., 2021). Electron microscopy and immuno-FISH experiments using female and male meiotic chromosome spreads estimated the loop size on several autosomes to be about 2.2 mm in leptotene, and about 6 μm in pachytene (Novak et al., 2008; Kauppi et al., 2011). Considering an estimated DNA density of about 40 kb to 94 kb/μm of chromatin loops (Moens and Pearlman, 1988; Kauppi et al., 2011; Ito et al., 2014), loop size would be expected to be smaller than what deduced from HiC data: about 250–500 Kb. However, as mentioned above, Immuno-FISH experiments on chromosome spreads have several limitations and do not take into account loop extension in space. This could be one of the reasons, why cytological measures do not fit Hi-C data. These differences need to be investigated more thoroughly. Both REC8 and RAD21L could participate in loop extrusion, and loop boarders could be potentially defined by CTCF sites and sites of meiotic DSBs. Indeed, DSBs in vegetative cells are enriched in cohesins (Strom et al., 2004; Unal et al., 2004) and DSBs have recently been shown to anchor cohesins at loop bases both in human cells (Arnould et al., 2021) and *S. cerevisiae* (Piazza et al., 2020).

### The Role of Axis Components in Homolog Pairing Before Meiotic Recombination

In several studies in *S. cerevisiae* and mammals, homolog pairing is detected even in the absence of meiotic recombination (Cha et al., 2000; Boateng et al., 2013; Ishiguro et al., 2014). Obviously, this is also a well-established property of meiosis in species (e.g., *D. melanogaster* and *C. elegans)* where pairing and homologous synapsis take place before DSB formation (Gerton and Hawley, 2005). The molecular mechanism of this pairing process is unknown and different species may adopt distinct strategies. In the context of the meiotic chromosome structure, one hypothesis is that cohesins mediates homologous pairing. This is based on two observations. First, in mouse spermatocytes, REC8 and RAD21L form distinct foci that can be at similar positions on both homologs. Each homolog would then have a specific bar code defined by these cohesin complexes, and this would allow a specific interhomolog interaction (Ishiguro et al., 2011). Second, recombination-independent (i.e., in *Spo11* mutants) homologous pairing detected by immuno-FISH assays (Boateng et al., 2013; Ishiguro et al., 2014), is defective in *Rad21L*^–/–^ but not in *Rec8*^–/–^ mice, suggesting a direct or indirect role for the RAD21L cohesin complex (Ishiguro et al., 2014). In *S. pombe*, where recombination-independent pairing has been analyzed in detail (Ding et al., 2012, 2019), cohesins are required (Ding et al., 2016). This could indicate a direct role of cohesins in pairing, or a more indirect role through the establishment of proper chromosome organization.

### The Axis Role in Meiotic Recombination

The chromosome axis is a platform for regulating meiotic recombination and specially to regulate DSB formation, interhomolog bias and CO formation during DSB repair. The contribution of the axis structure to DSB repair involves several additional components, namely the proteins that organize the central element of the synaptonemal complex for synapsis starting at zygonema. This aspect of the regulation of DSB repair and synapsis involves many factors and molecular pathways, will not be reviewed here, and only a few points will be highlighted below.

#### Impact of the Axis Structure on DSB Activity

Double-strand breaks repair takes place in the context of the chromosome axis, as shown by the many studies that described the axial localization of proteins involved in the early steps of DSB repair, such as RPA, RAD51, and DMC1 (Bishop, 1994). Meiotic DSBs might thus be introduced on genomic DNA sequences localized near the axis. It is thought that this is ensured, at least in part, by the axis-specific localization of the RMM complex, or Rec114/Mei4/Mer2 (REC114/MEI4/IHO1 in mammals), a protein complex that is essential for DSB activity. Indeed, the axis-associated proteins, cohesins, HORMADs, SYCP2 and SYCP3 in mammals, constitute a basis for loading the RMM complex (Winters et al., 2014; Kumar et al., 2015; Stanzione et al., 2016; Bhattacharyya et al., 2019; Papanikos et al., 2019; Acquaviva et al., 2020; Fujiwara et al., 2020; Figure 4). Such control also implies the tethering of potential DSB sites to the axis, as proposed by N. Kleckner (Blat et al., 2002). Indeed, in *S. cerevisiae*, DNA sequences that are constitutively axis-associated and interact with Rec8 or Red1 are not DSB sites, possibly because the DNA is not accessible to Spo11. In fact, it has been suggested that Rec8 antagonizes DSB formation in yeast and *A. thaliana* (Ito et al., 2014; Nambiar and Smith, 2018; Lambing et al., 2020). As described earlier (see section “The Meiotic Cohesin Complex, With the Specific α-Kleisin Rec8”), in *S. cerevisiae*, Rec8 is enriched near the 3′ ends of convergent genes. Thus, the potential DSB sites, which are promoter regions and have accessible chromatin, are brought, or stabilized to the axis thanks to the partly characterized interactions of H3K4me3 with Spp1 and of Spp1 with Mer2 (Acquaviva et al., 2013; Sommermeyer et al., 2013; Adam et al., 2018). How the RMM complex is localized remains to be understood and the interaction detected between HORMAD1 and IHO1 (Stanzione et al., 2016) and between Hop1 and Mer2 (Rousova et al., 2020) is one determinant for this localization.

Disrupting or changing Hop1 or Red1 localization directly by mutations or indirectly by altering cohesin localization has a direct consequence on DSB activity. This has been shown in *S. cerevisiae*, with changes of DSB localization in the Rec8 mutant (Kugou et al., 2009; Sun et al., 2015). When Red1 or Hop1 is disrupted, the overall DSB activity is slightly or strongly reduced, respectively (Mao-Draayer et al., 1996; Schwacha and Kleckner, 1997). Similarly, DSB activity is reduced in *Hormad1*^–/–^ mice (Daniel et al., 2011). Moreover, the chromosome axis has additional roles by ensuring feedback controls of DSB activity *in cis*. DSB formation leads to ATM (Tel1) kinase activation that negatively regulates locally and remotely DSB activity (Garcia et al., 2015). This local effect is likely to be explained by the chromosomal organization around DSB sites. It has been proposed that chromatin loops are physical units than can be targeted by the DSB machinery. This is consistent with the observation that increasing loop density, such as in the mouse PAR region, increases the potential for DSB activity (Kauppi et al., 2011). Long distance effects of ATM regulation could involve axis and chromatin components. One well known ATM dependent-chromatin modification that is observed also away from DSB sites (50 Kb in yeast, a few Mb in mammals) is the phosphorylation of the H2A variant H2AX, named as gH2AX (Mahadevaiah et al., 2001). As reported in somatic cells upon DSB induction, gH2AX spreads over large domains around the DSB sites, but its spreading is constrained at TADs in a process that requires cohesin-mediated loop extrusion (Caron et al., 2012; Arnould et al., 2021).

On chromosome axes, the Hop1/HORMAD1 protein also plays a role in inhibiting DSB activity and in checkpoint signaling. One pathway for this regulation is mediated by the displacement of Hop1/HORMAD1 from the chromosome axis upon synapsis triggered by homologous DSB repair. Hop1/HORMAD1 displacement leads to delocalization of the RMM complex, and thus downregulation of DSB activity. Additional regulations for signaling meiotic progression involve axis interactions and the ATR kinase (Daniel et al., 2011; Widger et al., 2018; Dereli et al., 2021).

#### Impact on DSB Repair

Although regulation of DSB repair is a wide and complex process beyond the scope of this review, few notable observations link chromosome axis to DSB repair. Hop1/Red1 ensures the bias of DSB repair toward the homolog, at the expenses of inter-sister repair. Indeed, Hop1/Red1 counteracts the activity of Rec8 that promotes sister chromatid cohesion (Kim et al., 2010). The underlying mechanism is not known, but it may occur through the regulation of one or several DSB repair steps, such as strand invasion, the activity of strand exchange proteins, or 3′ end extension. Interestingly, this also implies the uncoupling of the two DSB ends, one to be engaged in interaction with the sister chromatid, the other one excluded from this interaction. Additional chromosome axis structural components that are implicated in DSB repair through formation of the central element of the synaptonemal complex upon synapsis, play important roles in the decision of repair toward CO or non-CO. This control ensures that each homolog pair has undergone at least one CO.

## Concluding Remarks

### Main Properties and Functions

– The highly specialized organization of meiotic chromosomes is put in place at the onset of meiotic prophase I with some early components already bound to chromatin during the meiotic S phase.
– This organization is characterized by chromatin loops anchored to a protein axis. The main structural components and their roles are evolutionarily conserved. These components include cohesins, meiotic HORMA domain proteins, and structural components (Red1 in *S. cerevisiae*, SYCP2 in mammals, HTP-1/2, -3, HIM-3 in *C. elegans*, ASY3 in plants). Cohesin complexes recruit the other components, although these may also bind to chromatin independently from cohesins. Cohesin-mediated recruitment might be important to initiate axis formation. As shown *in vitro*, some axis structural components can multimerize and form filaments (e.g., Red1, SYCP2 and ASY3 with their partners) and are thus predicted to promote elongation of the axial structure. This structure is not static, and loop size and axis length can vary in an anti-correlated manner.
– Several meiotic specific cohesin complexes can co-exist in several species and differ by their α-kleisin subunits. The somatic cohesin complex may also be expressed in meiocytes. Those cohesin complexes achieve several functions, sister chromatid cohesion, loop organization and partner choice for DSB repair. The RAD21L (or COH-3/4) cohesin complex has no detectable sister chromatid cohesion activity. The function of this complex remains to be understood, but it might promote chromatid interactions during meiotic DSB repair.
– The axis forms a platform for loading other components, among which there is the RMM (Rec114, Mei4, Mer2/IHO1) complex that is essential for meiotic DSB formation. The axis-component Hop1/HORMAD1 plays a central role in turning on and off DSB activity with several additional consequences for recombination and meiotic progression. Hop1/HORMAD1 are regulated by conformational changes, mediated by Pch2/PCH2.
– Loops define domains that contribute to DSB activity regulation.
– Anchoring DSB formation to the chromosome axis allows organizing DSB repair regulation, specifically for controlling the inter-homolog bias and the crossover/non-crossover choice.

### Future Challenges

What are the determinants of chromatin loop organization? Although cohesins are likely to promote the formation of these loops, how their activities are regulated, how loops are spatially organized along the axis, what is the variability in loop size, and how are loops from the two sisters and from the two homologs organized remain to be determined.

Does this organization participate in homolog pairing and if yes, how?
How are these structural components organized relative to the genomic DNA sequence?
What are the role and activity of the meiotic RAD21L cohesin complex?
Does a loop define a domain for DSB activation? How are the borders defined?
How does the loop/axis from one homolog interact with the loop/axis from its partner upon DSB formation?

## Author Contributions

Both authors listed have made a substantial, direct and intellectual contribution to the work, and approved it for publication.

## Conflict of Interest

The authors declare that the research was conducted in the absence of any commercial or financial relationships that could be construed as a potential conflict of interest.

## Glossary

Axial element: the proteinaceous structure that provides an anchor for loop formation and forms at leptonema.
Lateral element: when the axial elements from two homologs engage into synapsis, a central element is formed, and the axial elements are named lateral elements.
Synaptonemal complex: the tripartite structure, a central element and two lateral elements that forms upon synapsis. Initiation of synapsis defines the zygotene stage.
Synapsis: the event associated with synaptonemal complex formation.
DSB: DNA double strand breaks.
Pre-leptotene: stage that includes the meiotic S phase.
TAD: topologically associated domains.
PAR: the region of homology between the X and Y sex chromosomes defined as the pseudo-autosomal region.
ChIP-seq: chromatin immunoprecipitation followed by next generation sequencing.
HiC: genome wide chromosome conformation capture.
